# ESR Essentials: trauma team and the role of Interventional Radiology—practice recommendations by the Cardiovascular and Interventional Radiological Society of Europe

**DOI:** 10.1007/s00330-026-12440-8

**Published:** 2026-03-24

**Authors:** Antonio Bulum, Bora Peynircioglu, Dimitrios K. Filippiadis, Philippe L. Pereira, Florian Wolf

**Affiliations:** 1 Department for Diagnostic and Interventional radiology, Section for interventional and cardiac radiology University Hospital Dubrava, Dubrava, Croatia; 2 Professor of Radiology, Hacettepe University, Ankara, Turkey; 3 Associate Professor of Diagnostic and Interventional Radiology, 2nd Department of Radiology, University General Hospital “ATTIKON”, Medical School, National and Kapodistrian University of Athens, Athens, Greece; 4 Professor of Radiology, Director Center of Radiology, Minimally Invasive Therapies and Nuclear Medicine, SLK-Kliniken GmbH, Academic Hospital of Ruprecht-Karls-University, Heidelberg, Germany; 5 Associate Professor of Radiology, Vice Director of the Division of Cardiovascular and Interventional Radiology, Department of Biomedical Imaging and Image-Guided Therapy, Medical University of Vienna, Vienna, Austria

**Keywords:** Trauma, Radiology, Interventional Radiology, Multidisciplinary collaboration, Emergency medicine

## Abstract

**Abstract:**

Trauma-related hemorrhage remains a leading cause of preventable death, requiring rapid diagnosis and timely intervention. Interventional Radiology (IR) plays a central role in the management of non-compressible bleeding, especially in solid organ injuries and pelvic trauma. This article outlines three key recommendations for integrating IR into trauma care. First, IR must be embedded in trauma teams with 24/7 availability at Level I trauma centers and structured access at Level II and III centers. Second, whole-body contrast-enhanced CT should be performed in hemodynamically stable or initially unstable but responsive patients, with immediate embolization when active extravasation or pseudoaneurysm is identified. Third, standardized embolization protocols and immediate access to essential materials—such as coils, plugs, liquid embolics, and stentgrafts—are critical for effective bleeding control. These recommendations are supported by current European guidelines and selected observational studies. To implement this guidance, trauma centers should develop IR-inclusive algorithms, define access pathways, and maintain trauma-ready IR inventories. Close collaboration between radiologists, surgeons, and emergency teams is essential to optimize patient outcomes and ensure timely intervention.

**Key Points:**

- *Interventional radiologists should be fully integrated into trauma teams with 24/7 availability in Level I centers.*
- *Whole-body contrast-enhanced CT should be performed in stable trauma patients, followed by immediate embolization when active bleeding is detected.*
- *Standardized protocols and materials must be in place to ensure rapid and effective embolization in trauma-related hemorrhage*.

## Key recommendations

The grading follows the World Society of Emergency Surgery (WSES) and AAST system, where the Level of Evidence reflects the quality of supporting data, and the Strength of Recommendation reflects the degree of clinical endorsement.

1. Interventional Radiologists should be integrated into the trauma team with 24/7 availability in Level I centers.

Level of Evidence: III, Strength of recommendation: B

- IR must be involved in trauma team activation and decision-making.
- Angiosuites should be located in the vicinity of the trauma bay to reduce delays.
- Level II–III centers require structured IR access or transfer protocols.

2. Perform whole-body contrast-enhanced CT in stable or stabilizable trauma patients to guide IR intervention.

Level of Evidence: II, Strength of recommendation: A

- Use plain/unenhanced, arterial and portal venous phases to detect bleeding.
- Initiate embolization promptly if active extravasation in the arterial phase or pseudoaneurysm is present.
- Hybrid emergency rooms (ERs) allow seamless CT-to-IR workflow.

3. Standardize embolization protocols and material availability in trauma IR.

Level of Evidence: IV, Strength of recommendation: B

- Ensure rapid access to coils, plugs, liquid agents, stent-grafts and resuscitative endovascular balloon occlusion of the aorta (REBOA).
- Define local protocols for organ-specific strategies, including criteria for non-operative management (NOM).
- Provide IR-specific trauma training and equipment kits.

## Introduction

Trauma is one of the leading global causes of death in patients under 45, with uncontrolled bleeding as the most preventable cause of early mortality. Rapid diagnosis and timely hemorrhage control are essential to improve outcomes. While trauma surgery remains central in many trauma protocols, Interventional Radiology (IR) has become increasingly important in managing non-compressible bleeding and solid organ injuries through minimally invasive procedures [1].

The importance of endovascular techniques in traumatic hemorrhage was formally recognized at a European level more than a decade ago with the publication of the CIRSE quality improvement guidelines for endovascular treatment of traumatic hemorrhage in 2012 [2]. Since then, advances in trauma imaging, embolization techniques, materials, and multidisciplinary trauma care have further expanded the role of IR.

However, access to IR in trauma care remains inconsistent across European centers. No formal European-wide standards currently define when and how IR should be integrated into trauma workflows. Trauma centers vary in their capability to provide around-the-clock IR support depending on their level of care, while clinical pathways often lack clearly defined processes for timely IR activation [3].

This document provides practical, evidence-based recommendations to guide the integration of IR into trauma care. It identifies key clinical scenarios where IR provides benefit, highlights organizational requirements for timely access, and supports implementation through examples derived from current guidelines and best practice. This is not a systematic review of the literature; a number of separate literature searches were performed, and non-English studies and case reports were excluded. All references of the articles obtained were also evaluated for additional information. These recommendations aim to standardize the role of IR across trauma systems and reduce delays in life-saving care.

## **General aspects of IR in trauma management**

### European Trauma Center Classification and IR responsibilities

In Europe, trauma centers are stratified according to their capabilities and available infrastructure, which directly determines the involvement of Interventional Radiology (IR) in acute trauma management [3].

- Level I centers provide comprehensive, 24/7 multidisciplinary trauma care, including continuous access to Interventional Radiology, surgery, and intensive care units.
- Level II centers can manage and stabilize many trauma cases, but may lack on-site IR or intensive care unit (ICU) support at all times.
- Level III centers are primarily responsible for the initial stabilization of trauma patients before arranging transfer to higher-level facilities.

IR is considered essential in Level I centers and is expected to be fully integrated into emergency protocols. In Level II centers, IR should be readily available through structured call systems, while Level III centers must ensure efficient coordination for the rapid transfer of patients requiring IR services. The presence and availability of IR can be decisive for effective hemorrhage control and organ preservation, particularly in patients with non-compressible bleeding.

### Interventional Radiology in the trauma setting

Interventional Radiology (IR) has become an essential pillar of modern trauma and emergency care, providing minimally invasive, image-guided solutions for managing life-threatening conditions. By combining advanced imaging with endovascular techniques, IR facilitates rapid diagnosis and targeted treatment, often reducing the need for open surgery and improving clinical outcomes [4]. Early involvement of an Interventional radiologist is strongly recommended in trauma cases where IR may offer a therapeutic benefit—particularly in managing vascular injuries and solid organ trauma. Optimal trauma care requires a multidisciplinary team (MDT) approach that includes emergency and intensive care specialists, diagnostic radiologists, interventional radiologists, anesthesiologists and trauma/orthopedic surgeons [5].

In modern trauma care, emergency physicians are the first point of contact and play a pivotal role in the initial assessment and stabilization of patients [6]. Assessment should be guided by the Advanced Trauma Life Support (ATLS) protocol, ensuring that life-threatening conditions are identified and addressed in a structured, prioritized manner [7].

Following stabilization, diagnostic radiologists become involved to evaluate the trauma and enable triage. Imaging modalities are conventional radiography (CR), US including FAST evaluation ( = Focused Assessment with Sonography for Trauma) and MSCT ( = multislice computed tomography). While CR and US retain value in specific indications—particularly in blunt abdominal trauma—total-body MSCT is now the gold standard in evaluating polytrauma patients due to its speed and diagnostic precision [8]. MRI, by contrast, has limited utility in acute trauma because of longer scan times and lower availability, as well as low diagnostic value for acute bleedings.

A notable advancement in imaging-driven trauma care is the hybrid emergency room (ER) concept, developed in Japan. These units integrate CT, Interventional Radiology (IR), and surgical capability within a single resuscitation space. As demonstrated by Kinoshita et al, this model allows for immediate whole-body imaging, collaborative interpretation by surgeons and radiologists, and seamless transition to angiographic or surgical intervention—all without relocating the patient [9]. This integration improves workflow efficiency and has been associated with better survival outcomes in urban trauma centers. While this model is not yet widely implemented outside Japan, it represents an important future perspective for optimizing trauma workflows and underscores the operational and clinical value of early IR activation.

Once diagnostic data are available, the MDT formulates an individualized treatment plan [10, 11]. One of the first critical decisions involves determining whether operative or non-operative management ( = NOM) is appropriate [12]. Operative intervention is generally required in cases of hemodynamic instability, ongoing hemorrhage, or structural compromise such as hollow-organ perforation or vascular disruption that cannot be controlled by endovascular means. Failure of conservative approaches refers to secondary clinical deterioration—e.g., persistent bleeding with increasing hemodynamic instability or expanding hematoma—after an initial attempt of NOM. Conversely, non-operative strategies are increasingly favored for patients with blunt trauma and stable hemodynamics, where Interventional Radiology plays a central role in controlling active bleeding and preventing surgical exploration [13]. Almost exclusively, IR should be prepared to stop acute hemorrhage as part of the initial management pathway. Within the MDT, direct consultation between diagnostic and Interventional radiologists enhances the treatment strategy and reduces trauma-related morbidity and mortality [14].

IR procedures are done on a broad basis in acute and severe trauma patients [15–17]. In Level I trauma centers, where a 24/7 Interventional Radiology service is a core requirement, these procedures represent an essential component of hemorrhage control. The frequency of these procedures compared to surgical alternatives is highly variable between different centers and countries and mostly depends on the presence and 24/7 availability of an Interventional Radiology service.

Clinical evidence regarding the management of hemodynamically unstable patients remains inconclusive. However, more recent studies [18–20] have demonstrated a clear advantage, particularly when Interventional Radiology is available on a 24/7 basis. For hemodynamically stable patients, CT-based diagnosis followed by selective embolization has become an established standard of care, showing high technical success rates and favorable outcomes across multiple organ systems [16–20].

The 2023 edition of the European guideline on the management of major bleeding and coagulopathy following trauma provides specific recommendations concerning the role of Interventional Radiology [21]. A Shock Index—defined as the ratio of heart rate to systolic blood pressure—greater than 1 is highlighted as an early indicator for considering IR involvement. For hemodynamically stable patients, immediate whole-body CT is recommended to facilitate comprehensive injury assessment. The guideline recommends considering intraoperative cell salvage (ICS) in cases of severe bleeding into the abdomen, pelvis, or thorax; when applied within the angiosuite or hybrid trauma room, ICS can be performed alongside damage-control embolization to reduce allogeneic transfusion requirements. This aligns with broader patient-blood-management guidance [22] and evidence from surgical and trauma settings demonstrating decreased exposure to allogeneic blood products and favorable cost-effectiveness [23, 24].

Transcatheter arterial embolization (TAE), alone or in combination with REBOA, has been emphasized in recent consensus recommendations as an effective component of multidisciplinary trauma management in hemodynamically unstable patients [25, 26]. Both the Italian and the WSES-AAST guidelines highlight that when Interventional Radiology is promptly available, embolization enables rapid hemorrhage control and contributes to improved survival. Although prospective comparative evidence remains limited, these expert statements underscore the feasibility and life-saving potential of endovascular bleeding control within structured trauma workflows.

A fully equipped angiography suite with rapid access is essential, including high-performance fluoroscopy, dedicated angiography-trained staff, and immediate availability of microcatheters, guiding catheters, and embolic materials such as coils, plugs, particles and liquids as embolization tools. These resources allow targeted vessel occlusion with minimal delay and are critical for the success of TAE in the acute trauma setting.

In addition to clinical outcome data, the importance of Interventional Radiology as an integral member of the multidisciplinary trauma team (MDT) has been highlighted. Okada et al demonstrated improved survival in hemodynamically unstable trauma patients when IR was directly involved in the MDT. Early IR participation was associated with faster decision-making and shorter procedure times, underscoring the organizational value of IR integration beyond the technical procedure itself [20].

### Bleeding management

**Fig. 1:**
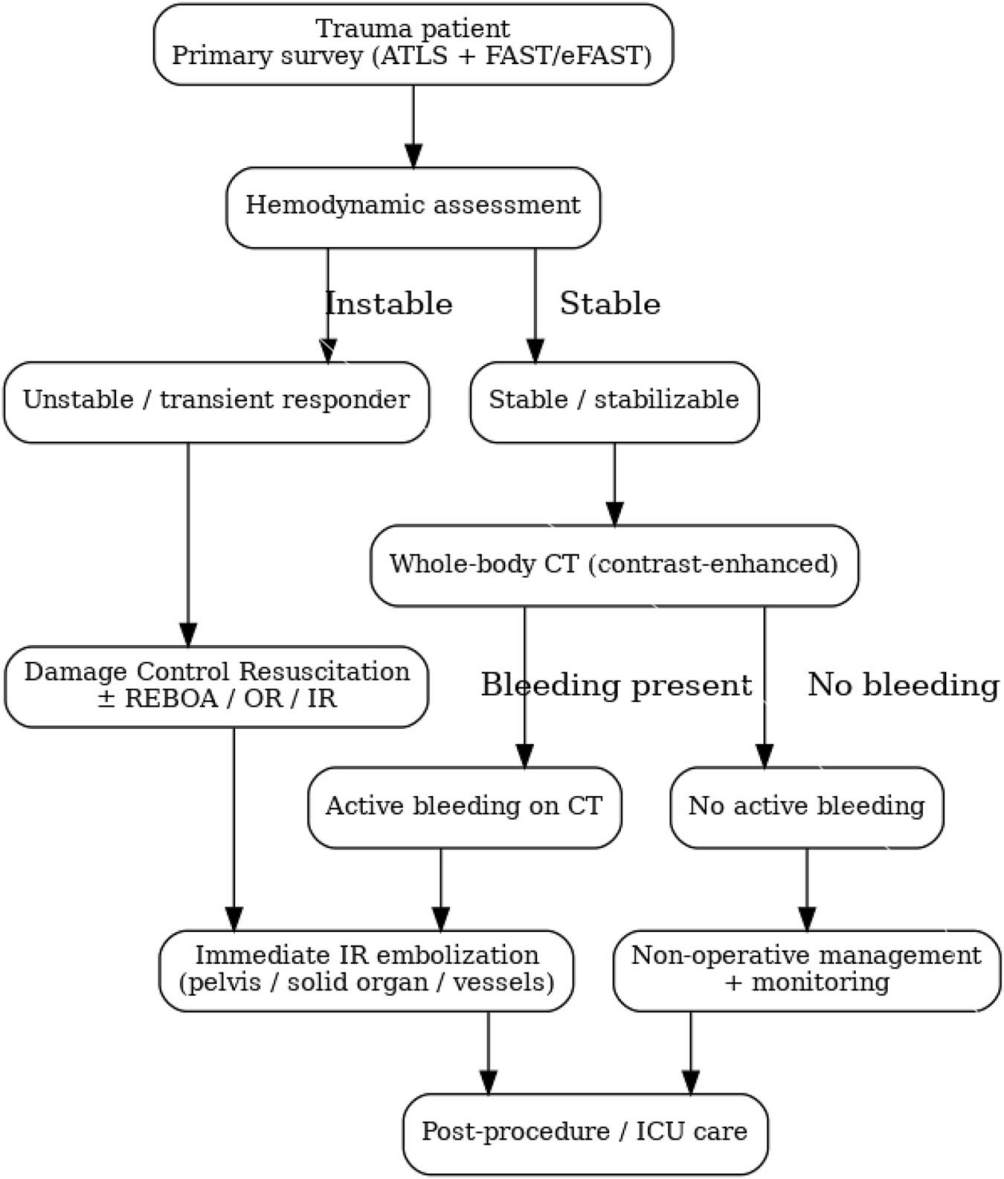
Simplified trauma algorithm with IR integration. Unstable patients undergo immediate resuscitation and IR/surgical consideration, while stable or stabilizable patients proceed to whole-body CT with embolization if active bleeding is detected. ATLS, advanced trauma life support; FAST, focused assessment with sonography for trauma; eFAST, extended focused assessment with sonography for trauma; REBOA, resuscitative endovascular balloon occlusion of the aorta; OR, operating room; IR, Interventional Radiology; CT, computed tomography; ICU, intensive care unit

Vascular injuries causing profuse arterial bleeding are the major and most urgent indication for Interventional Radiology (IR) in trauma. These include arterial lacerations, transections, pseudoaneurysms, or active extravasation, where IR enables rapid, targeted, and life-saving hemorrhage control. Less frequently, IR is applied for vascular occlusions from traumatic dissections, particularly in visceral vascular injuries. Overall, the principal role of IR in trauma lies in the prompt management of life-threatening bleeding. (Fig. 1).

## Specific trauma management

### Pelvic fractures

Pelvic fractures, especially when accompanied by arterial bleeding, carry a high risk of death, with mortality rates reaching up to 50% in patients with open pelvic fractures.

In hemodynamically stable pelvic fractures without evidence of major arterial bleeding, orthopedic stabilization with compressive pelvic binders can be sufficient to control venous or cancellous bone hemorrhage. According to the EAST and WSES trauma guidelines [27, 28], binders are particularly effective in anterior–posterior compression (APC) injuries, where they reduce pelvic volume and tamponade low-pressure venous bleeding. In contrast, patients who remain unstable despite mechanical stabilization, or in whom imaging demonstrates active arterial extravasation, require urgent endovascular management. In such cases, Interventional Radiology represents the primary life-saving approach, while surgical pelvic packing should be considered only when IR is unavailable or has failed to achieve hemostasis [20].

Angioembolization has proven highly effective in controlling hemorrhage, with success rates exceeding 85% when performed promptly. Commonly targeted vessels include branches of the internal iliac artery, such as the superior gluteal or obturator arteries. Depending on the bleeding pattern and vessel caliber, coils, gelfoam, or liquid embolic agents may be used—either alone or in combination—to achieve rapid and durable hemostasis. Bilateral embolization may be required in some cases to achieve hemostasis, and early intervention is critical to improving outcomes [29, 30].

### Extremity fractures

In extremity trauma, major hemorrhage requiring Interventional Radiology (IR) is uncommon compared with abdominal or pelvic bleeding. In most cases, hemorrhage can be effectively managed with direct compression, tourniquet application, or surgical repair. IR is generally reserved for selected situations where bleeding persists despite these measures, or where the injured vessel is deep, inaccessible, or associated with complex fractures. Typical indications include active arterial extravasation, pseudoaneurysm formation, arteriovenous fistula, or focal arterial transection visible on CT angiography. When performed, superselective embolization, balloon occlusion, or stent-graft placement can achieve rapid hemostasis while preserving collateral perfusion and minimizing the risk of limb ischemia.

Although registry data confirm that the vast majority of trauma angiographies involve the abdomen or pelvis—reportedly over 80%—specific data on the frequency of extremity interventions are lacking. Current evidence and guidelines therefore support IR as a targeted, adjunctive therapy in carefully selected cases, rather than a routine first-line approach for extremity bleeding [5, 7].

### Solid organ injuries

The American Association for the Surgery of Trauma (AAST) developed the Organ Injury Scale (OIS) to classify the severity of organ injuries. First published in 1989, the system now includes grading criteria for 32 different organ systems, ranging from Grade I (minor injury) to Grade V (severe injury) [31]. Updated versions have incorporated radiologic findings, such as signs of active bleeding—recognized as key predictors of failed NOM. For example, the splenic injury scale was revised in 2018 to reflect these changes.

Generally, based on AAST grading, low-grade injuries (Grades I–II) are amenable to conservative management without the need for invasive intervention. In contrast, higher-grade injuries (Grades III–V) are more likely to require surgical and/or IR treatments [32].

The AAST recommends multiphase computed tomography (MSCT) as the diagnostic gold standard, especially for hemodynamically unstable patients. Native, arterial, and portal venous phases are generally advised for detecting bleeding. Alternatively, virtual non-contrast (VNC) imaging may be used instead of a native scan.

Transarterial embolization (TAE) is a cornerstone of NOM for injuries to solid organs such as the liver, spleen, and kidneys [2]. By targeting active bleeding sites identified on contrast-enhanced CT, TAE enables precise hemostasis while preserving organ function. In the Society of Interventional Radiology (SIR) Position Statement on Endovascular Intervention for Trauma, this revision was highlighted, and the role of embolization in improving organ salvage and reducing NOM failure rates, particularly in high-grade injuries (Grades IV and V), was emphasized [11].

#### Liver injuries

The AAST classification system divides liver injuries into five grades based on the depth and extent of parenchymal disruption and vascular involvement:

- Grade I: Capsular tear < 1 cm depth or subcapsular hematoma < 10% of surface area.
- Grade II: Parenchymal laceration 1–3 cm depth and ≤ 10 cm length, or contained hematoma 10–50% of surface area.
- Grade III: Laceration > 3 cm depth or expanding hematoma > 50% of surface area.
- Grade IV: Parenchymal disruption involving 25–75% of a hepatic lobe or 1–3 Couinaud segments within a single lobe.
- Grade V: Parenchymal disruption > 75% of a hepatic lobe, or juxtahepatic venous injury (e.g., retrohepatic inferior vena cava (IVC) or major hepatic vein).
- Grade VI: Hepatic avulsion (rare, usually fatal).

Management of liver trauma depends largely on the patient’s hemodynamic stability and imaging findings. In hemodynamically stable patients, treatment is stratified based on the presence or absence of contrast extravasation or pseudoaneurysm on imaging rather than solely on the injury grade. Patients with low- to moderate-grade injuries—AAST Grade I-III and without active bleeding can typically be managed conservatively with clinical and laboratory monitoring, along with follow-up imaging when appropriate. Higher-grade lesions—Grade IV and V carry a greater risk of ongoing hemorrhage and often require intervention. If a contrast blush or vascular abnormality is identified, transarterial embolization is indicated as a first-line treatment. In cases where bleeding persists despite embolization or the patient remains unstable, damage-control surgery with hepatic packing is recommended as the initial operative step, whereas liver resection is reserved for non-salvageable parenchymal destruction or failed packing. According to the WSES 2020 liver-trauma guidelines, treatment outcomes depend on patient condition and institutional resources, particularly the immediate availability of experienced interventional radiologists and 24/7 angiographic capability [33].

In hemodynamically unstable patients (AAST Grades IV–V), immediate surgical intervention is required. Resuscitative Endovascular Balloon Occlusion of the Aorta (REBOA or REBOA-C) may be used as a last-resort measure in order to stabilize the patient. After initial management, all patients are transferred to the ICU for continued monitoring and care [31].

The liver is among the most commonly injured abdominal organs. Studies demonstrate that TAE achieves hemostasis in over 90% of hemodynamically stable patients with high-grade liver injuries, reducing the need for surgery. Techniques range from superselective embolization using microcatheters to “scatter” embolization of an entire hepatic lobe [33].

#### Splenic injuries

The AAST classification system divides splenic injuries into five severity grades.

- Grade I includes subcapsular hematomas involving less than 10% of the organ surface and capsular lacerations less than 1 cm deep.
- Grade II refers to subcapsular hematomas affecting 10–50% of the surface or lacerations 1–3 cm deep.
- Grade III describes subcapsular hematomas involving more than 50% of the surface or lacerations deeper than 3 cm.
- Grade IV includes parenchymal injuries involving segmental arteries or the central venous trunk.
- Grade V represents vascular injury with complete devascularization or hilar vessel avulsion.

In patients with minor splenic injuries (AAST I–II), conservative management with clinical and imaging follow-up is usually sufficient. Moderate injuries (AAST III) are evaluated by contrast-enhanced CT, which detects active contrast extravasation or pseudoaneurysm as indicators of arterial bleeding. In these cases, angiography with transcatheter embolization is recommended as first-line therapy. Two main endovascular strategies exist: proximal splenic artery embolization, which lowers arterial pressure within the spleen and promotes spontaneous hemostasis while preserving collateral flow, and distal (selective) embolization, which directly occludes the bleeding branch when active extravasation is identified. The proximal approach, typically achieved by coil occlusion of the main splenic artery, has been shown to significantly reduce rebleeding rates and transfusion requirements [34, 35]. Compared with liver and renal trauma, splenic injuries are often more insidious, as bleeding may intermittently cease or recur; therefore, close hemodynamic and imaging surveillance remains essential even after successful embolization. Surgery is reserved for hemodynamically unstable patients or extensive splenic disruption, while IR remains the preferred approach in most other cases.

#### Renal injuries

Kidney Injury Classification (AAST):

The AAST scale classifies renal trauma from Grade I (minor) to Grade V (severe).

- Grade I: Contusions or subcapsular hematomas without laceration.
- Grade II: Lacerations < 1 cm deep without urinary extravasation.
- Grade III: Lacerations > 1 cm deep without involvement of the collecting system.
- Grade IV: Lacerations extending into the collecting system or injuries to segmental arteries.
- Grade V: Shattered kidney or complete vascular avulsion.

In hemodynamically stable patients with CT-confirmed renal injuries (AAST I–V), conservative management is generally recommended. If active bleeding is present, selective embolization is the preferred treatment with the advantage of preserving renal function. If initial embolization fails, a second attempt is advised before considering surgery, as reintervention success rates are comparable to primary embolization (63–100%) [25].

Traumatic injury to the renal artery, including dissection or occlusion, is a rare but serious consequence of blunt or penetrating trauma that may lead to renal ischemia and infarction. Endovascular revascularization with stent placement or thrombectomy can be considered in hemodynamically stable or stabilized patients when imaging shows flow limitation or perfusion impairment. The optimal time window for successful revascularization is within approximately 4–6 h after injury; however, delayed intervention may still be justified when residual flow or partial perfusion is demonstrated on angiography [36–38].

### Vascular emergencies in trauma

IR plays a critical role in addressing vascular emergencies, such as arterial transections and dissection.

### Aortic and large vessel injuries

Traumatic aortic injury most commonly involves the thoracic aortic isthmus (≈ 90% of cases) after high-energy deceleration trauma. Less frequently, injuries occur in the ascending aorta (≈ 5–10%) or at the diaphragmatic hiatus (≈ 2–5%). The Society for Vascular Surgery (SVS) classification, adapted from the AAST system, defines four grades [39]:

- Grade I: Intimal tear
- Grade II: Intramural hematoma
- Grade III: Pseudoaneurysm
- Grade IV: Complete rupture

Grades I–II are managed non-operatively with blood-pressure control and serial imaging. Grades III–IV, representing pseudoaneurysm or rupture, require urgent endovascular repair (TEVAR) when anatomically feasible, while ascending aortic injuries are treated surgically. Early TEVAR is particularly indicated for thoracic transections with contained rupture or rapidly expanding pseudoaneurysm, whereas open repair is reserved for lesions not suitable for endovascular treatment or in centers without endovascular capability. Endovascular stent-grafting has markedly reduced mortality compared with open repair in descending thoracic aortic trauma [40].

### Musculoskeletal trauma and smaller vessel trauma

Interventional Radiology plays only a minor role in the management of bleeding associated with musculoskeletal trauma, since in most cases manual or external compression is sufficient to achieve hemostasis once coagulopathy has been corrected. IR becomes relevant only when bleeding persists despite compression or the injured vessel is deeply located or surgically inaccessible. In such situations, the principles of emergency embolization are universal—identify the bleeding source by angiography, achieve proximal and distal control, and ensure occlusion of potential collateral inflow.

If the affected artery can be sacrificed, coil or gelfoam embolization is typically performed. When arterial preservation is required, covered stents may be used selectively. The sandwich technique refers to embolizing both the proximal and distal segments of the injured vessel to prevent retrograde bleeding from collateral circulation. Although rare, these interventions are life- or limb-saving when indicated and follow the same endovascular principles regardless of anatomical site [41].

## Summary statement

Interventional Radiology (IR) is an essential component of modern trauma care, particularly in the management of non-compressible bleeding and solid organ injuries. Its early integration into trauma workflows improves patient outcomes by enabling rapid, minimally invasive hemorrhage control. IR should be an integral part of the multidisciplinary trauma team, with its role in the emergency setting clearly defined and guided by standardized trauma protocols.

This document provides three specific, evidence-informed recommendations: (1) IR must be available 24/7 and fully integrated into trauma teams, particularly at Level I trauma centers; (2) whole-body contrast-enhanced CT should be used in stable or stabilizable patients to guide timely IR intervention; and (3) centers must standardize embolization techniques and maintain immediate access to appropriate materials and equipment.

The recommendations offer a practical framework to support the structured and efficient use of IR in trauma care and should serve as a foundation for future guideline development and system-level improvements across Europe.

## Patient summary

Injuries from accidents or trauma can cause dangerous internal bleeding. Interventional Radiology (IR) is a medical subspecialty that uses imaging and provides minimally invasive treatments via small tools and tiny incisions to rapidly stop bleeding without the need for open surgery. This article explains how trauma teams can work better with IR doctors to treat patients faster and more safely. It recommends using whole-body scans to find bleeding early and having trained IR doctors ready at all times. These steps can help save lives and reduce the need for major surgery in emergency situations.

## Abbreviations

AAST: American Association for the Surgery of Trauma
CIRSE: Cardiovascular and Interventional Radiological Society of Europe
CR: Conventional radiography
CT: Computed tomography
ER: Emergency room
ICS: Intraoperative cell salvage
ICU: Intensive care unit
IR: Interventional Radiology
MDT: Multidisciplinary team
MSCT: Multislice computed tomography
NOM: Non-operative management
OR: Operating room
REBOA: Resuscitative endovascular balloon occlusion of the aorta
REBOA-C: Resuscitative endovascular balloon occlusion of the aorta–complete occlusion
TAE: Transcatheter arterial embolization
TEVAR: Thoracic endovascular aortic repair
US: Ultrasound
WSES: World Society of Emergency Surgery
WSES-AAST: World Society of Emergency Surgery—American Association for the Surgery of Trauma
